# Innovation in women’s health must be led by women

**DOI:** 10.1136/bmj.r2157

**Published:** 2025-10-16

**Authors:** Ebere Okereke

**Affiliations:** Chatham House, London, UK; Competing interests: The BMJ has judged that there are no disqualifying financial ties to commercial companies. The author declares the following other interests: I am chief programmes officer at Mohamed bin Zayed Foundation for Humanity but writing in a personal capacity. Further details of The BMJ policy on financial interests are here: https://www.bmj.com/sites/default/files/attachments/resources/2016/03/16-current-bmj-education-coi-form.pdf

## Abstract

Inclusive and accountable research and technology are essential for gender equity

After decades of progress, the world is quietly sliding backwards on gender equity.^1^ Gains that once seemed secure are being eroded by political backlash, economic pressure, and complacency. Investments in the emerging field of “women’s health innovation” are celebrated while the deeper inequities that make such innovation necessary remain intact.

Women’s health is still treated as a sub-sector, not as a foundational pillar of population health. Despite clear evidence that investing in women’s health improves outcomes for families and entire communities, global funding remains disproportionately low.^2^ Women continue to face diagnostic delays, limited access to specialised care, and the persistent exclusion of their lived experience from the design of health systems.^3^

This renewed attention to innovation offers an opportunity—but only if we redefine what innovation means and who drives it. Too often, innovation is equated with technology. Apps, wearables, and AI models are launched with claims to “empower women,” yet the fundamental barriers—social, structural, and political—remain unmoved. When innovation is defined by those who currently hold power, it risks becoming another mechanism that reinforces exclusion.

The BMJ Collection on Women’s Health Innovation (bmj.com/collections/womens-health-innovation ) invites us to look deeper. It spans topics from novel methods for incorporating sex and gender analysis in clinical trials to regulatory reforms to safeguard women’s privacy in femtech to embedding equity in innovation pathways using a decolonial feminist lens. Together, these articles highlight a fundamental truth: innovation in women’s health is not only about what we create but how and by whom it is created.

The under-representation of women in research remains a scandal. Trials still rely on data that understate sex differences in disease presentation and treatment response, show Peters and colleagues.^4^ The absence of women, particularly pregnant and older women, from research pipelines produces evidence that does not reflect real world patients. This is not a technical problem—it is a governance failure. Funders and regulators should stop rewarding studies that exclude women or treat gender analysis as optional.

Technology can accelerate progress, but only if it is inclusive and accountable. Digital health and AI tools for screening and diagnosis hold potential, yet many are designed and tested far from the communities they are meant to serve. In low resource settings, AI supported cervical cancer screening, for example, could be transformative—but only if it is built on local data, validated in real world conditions, and embedded in systems that guarantee follow-up and equity of access, as Linder and colleagues show.^5^

## Shifting the balance

Equity also depends on leadership. Who leads matters, Kedia and colleagues argue.^6^ Women’s health will not be transformed by systems still dominated by men. The global health ecosystem remains heavily skewed toward male decision makers, even in organisations focused on women’s rights and maternal health.^7^ When women do lead, they often face resistance, tokenism, or the expectation that their leadership should come at a discount. The result is predictable: the priorities of women are still being filtered through the lens of others.

We must confront this imbalance directly. Institutions should not just appoint women to advisory roles but give them the authority to allocate budgets, set research priorities, and define success.^8^ Funders must invest in female led organisations and research groups, especially in low and middle income countries, where innovation can be informed by women’s lived experience as well as expertise.^9^

Around the world, we are seeing the erosion of reproductive rights, the policing of women’s bodies, and the defunding of gender programmes. These reversals are not isolated—they are signals of a broader regression in how societies value women. The scientific community cannot be neutral on this. Journals, funders, and health institutions must defend evidence based policy, protect women’s rights to health and autonomy, and ensure that “innovation” does not become a cover for inequity.

Further, innovation in women’s health should be disruptive in the truest sense: it should challenge power, shift resources, and recognise that women’s health is shaped as much by social norms and economic policy as by medical technology.

The investment in women’s health research is welcome, but it must deliver differently. We cannot afford another cycle of well intentioned initiatives that overlook who benefits, who leads, and who is left behind. The test of progress will not be in the number of innovations launched but in whether women’s lives, health outcomes, and agency improve as a result. If innovation does not shift power, it is not innovation.

## Notes

Provenance and peer review: Commissioned; not externally peer reviewed.
